# Rethinking GWAS: how lessons from genetic screens and artificial intelligence could reveal biological mechanisms

**DOI:** 10.1093/bioinformatics/btaf153

**Published:** 2025-04-08

**Authors:** Dennis J Hazelett

**Affiliations:** Department of Computational Biomedicine at Cedars-Sinai Medical Center, West Hollywood, CA 90069, United States; Cancer Prevention and Control—Samuel Oschin Cancer Center, Los Angeles, CA 90048, United States

## Abstract

**Motivation:**

Modern single-cell omics data are key to unraveling the complex mechanisms underlying risk for complex diseases revealed by genome-wide association studies (GWAS). Phenotypic screens in model organisms have several important parallels to GWAS which the author explores in this essay.

**Results:**

The author provides the historical context of such screens, comparing and contrasting similarities to association studies, and how these screens in model organisms can teach us what to look for. Then the author considers how the results of GWAS might be exhaustively interrogated to interpret the biological mechanisms underpinning disease processes. Finally, the author proposes a general framework for tackling this problem computationally, and explore the data, mechanisms, and technology (both existing and yet to be invented) that are necessary to complete the task.

**Availability and implementation:**

There are no data or code associated with this article.

## 1 Genetic screens reveal the underlying biology of phenotype

Classic genetic screens in model organisms dissected the molecular basis of phenotypes ranging from developmental processes to behavioral patterns (Nurse and Hayles 2019). These screens mutagenized the germline and selected progeny of F1 self-crosses for lethality. Once established as stably propagating lines, carriers of lethal mutations were further screened in embryonic or larval stages for phenotypes that prevented them from reaching adulthood. Perhaps the best known of these screens was performed in the 1970s by Nüsslein-Vollhard and Wieschaus, who used this approach to systematically identify defects in the denticle patterns of the larval cuticle of *Drosophila*. They shared a Nobel Prize with the great geneticist Ed Lewis, whose seminal work on homeotic “monster” mutations inspired and made these screens possible (“The Nobel Chronicles” 2000). Their approach spawned new fields into existence and was subsequently replicated in other organisms, particularly yeast, roundworms, and vertebrates like zebrafish, each model having unique phenotypic and experimental advantages.

It took years and many subsequent experiments to work out details of tissue polarity, genes, and their relationships to each other. One thing that facilitated early efforts was that in model organisms, it was straightforward to map lethality relative to non-lethal markers such as eye color and wing shape to within tens of kilobases. Geneticists would use phage libraries to “positionally clone” the gene whose mutation resulted in lethality, and whose reintroduction was necessary and sufficient to rescue a phenotype.

Modern biologists have the reverse problem: they can detect the presence of many small-effect mutations with great sensitivity. They can know the exact sequence and its relationship to genomic annotations. But GWAS interpretation is complicated by population stratification, which can give spurious signals (Hellwege *et al.* 2017). Also, in contrast to forward lethal screens, most associations have low-penetrance, causing some to doubt their usefulness in the clinic. To circumvent this issue, epidemiologists use polygenic scores as a proxy for the cumulative effects of genotyping on the phenotype (Novembre *et al.* 2022, Yang *et al.* 2023). While low-penetrance has been used to question the clinical relevance of GWAS, from a perspective of disease etiology it does not matter. Prognostic value aside, understanding the root causes of disease is necessary for prevention and mitigation. For the majority of loci however, the task of assigning mechanisms and target gene identity is nearly intractable. Pleiotropic effects, in which the same allele causes more than one phenotype at the cellular or organismal level, can muddle the issue further (Watanabe *et al.* 2019).

There are two surprising facts about model organisms relevant to practitioners of human biology. One is that most phenotypes of interest to human medicine have cognates in model organisms. Yeast, flies, roundworms, mice, and fish have been screened for neuronal, wing, limb, and heart development, circadian rhythms, fertility, chemotaxis, thermotaxis, courtship, longevity, asymmetric cell division, natural immunity, addiction, anterior-posterior (AP) patterning, dorso-ventral patterning, gut chirality, chondrogenesis, wound healing, regeneration, cell division, migration, microtubule transport, epidermal–mesenchymal transition, and metamorphosis to name a few. Even though funding agencies long frowned upon them as “fishing,” screens have been the pace car of discovery in biology for decades. The open-ended nature of screens should not be considered hypothesis-free, but rather hypothesis-generating. After all, the goal of scientific studies is to rule out existing hypotheses or find better ones than currently exist. In fact, screens have also been used for hypothesis driven research, including conditional screens for modifiers of key genes and phenotypes.

The second, and perhaps most surprising fact for lay people, is that in nearly every case that researchers have compared phenotypes in model organisms and people, the critical molecular components, genes, and pathways are strikingly similar. The lessons learned from model organisms have illuminated cell biology and patterning, even where the similarities are likely to have been produced by convergence. In ways that seemed absurd a few decades ago, nature has yielded up secrets of human biology from the unlikeliest of places: from the teeming masses of soil, air, and sea. One of the most vivid examples of this is that the genetic machinery underlying specification and patterning of the compound insect eye is controlled by homologous transcription factors, *Pax6*/*eyeless*, that control the initiation, specification, and patterning of both vertebrate and invertebrate eyes (Halder *et al.* 1995, Tomarev 1997), despite fundamental differences in form and function. *Drosophila* homeobox transcription factors belonging to the Antennapedia (*ANTP*) class of homeobox genes control patterning of the AP axis and are arranged (incredibly) on the chromosome in the order in which they are expressed (Papageorgiou 2015). Comparative studies suggest that this cluster diversified by duplication, divergence, and selective retention of genes, elaborating the macro-morphic evolution of increasingly complex body plans and appendages (Hrycaj and Wellik 2016). Perhaps the strangest example of shared biology is that of fruit fly sleep homeostasis, whereby cells are governed by a highly coordinated molecular clock marking cyclical destruction and synthesis of the *period* and *timeless* genes. Homeostasis is regulated by neural circuits that respond to external stimuli, and, once set, remains faithful to the 24 h cycle [reviewed in Rosato *et al.* (2006)]. These mechanisms are highly conserved in vertebrates [reviewed in Patke *et al.* (2020)]. Overall, discoveries in model organisms have facilitated our increased understanding of human physiology and development at a molecular level.

## 2 Parallels and contrasts between genetic screens and human GWAS

In the decade following sequencing of the human genome, high throughput genotyping of single nucleotide polymorphisms made it possible to correlate genomic variation with phenotype at the population level. *Homo sapiens* thus became its own most powerful model organism (LaFramboise 2009). Surprisingly, early studies estimated >90% of disease-associated variation resides in non-coding regions, referred to in popular literature as “junk DNA” (Pennisi 2012). In retrospect, this observation makes sense for low-penetrance alleles that barely increase the likelihood of developing disease. This has led to a revolution in epigenomics research to better understand these regions. Assays to measure methylation at cytosines, post-translational modifications of histones to identify enhancers, promoters, and silenced regions, were carried out at scale. Leading the way were the ENCODE and Roadmap consortia, which cataloged experiments across cell lines and tissue samples with standardized protocols and antibodies (ENCODE Project Consortium 2012, Roadmap Epigenomics Consortium *et al.* 2015). The widely accepted computational approach was to identify the most relevant cell line or tissue and assign functionality to non-coding variants and gene targets by proximity, functional assay, inference from expression quantitative traits (eQTL) or chromatin interaction (Hazelett *et al.* 2016). Despite early skepticism (Bourgain *et al.* 2007, McClellan and King 2010, Crow 2011), GWAS has led to identification of thousands of common variants associated with susceptibility to disease. Subsequently, researchers of model organisms (Flint and Eskin 2012, Wangler *et al.* 2017) and even plants (Naake *et al.* 2024) have adopted GWAS to study variation and phenotype in natural and cultivated populations.

Though there are many important differences distinguishing GWAS from forward genetic screens, some parallels are unavoidable. For instance, both methods rely on correlation between a genotype and a phenotype. Both have random gene disruption. Both have recovered point mutations in coding and non-coding regions alike. Forward genetic screens can be biased toward different types of DNA sequence lesions from chemical and radiological exposure. Sequencing whole genomes of tumor and healthy tissues similarly has revealed characteristic signatures of different mutagenic agents in the environment (Alexandrov *et al.* 2020).

GWASs interrogate differences in phenotype between major and minor alleles of common, preexisting population variants. These variants are fundamentally different from tumor somatic mutations (ICGC/TCGA Pan-Cancer Analysis of Whole Genomes Consortium 2020) and induced lesions of genetic screens. However, most common variants arose randomly and expanded in human populations over time. Expansion occurred by genetic drift from founder effects and population bottlenecks, admixture, or gene flow from intermingling of previously isolated populations, and random mutations (The 1000 Genomes Project Consortium 2015, Bergström *et al.* 2020). Most variants are benign, with a small fraction being adaptive. Whatever their origin, they yield so slight an advantage that they become fixed in Hardy–Weinberg equilibrium, such that their effects are refractory to reproductive fitness. They are more or less uniformly distributed in the subject population, and give rise to phenotypes which manifest under the tightly defined parameters of GWAS, usually age and environmental factors. By comparison, screen mutations are also random and detected by assay under the tightly controlled parameters of the genetic screen.

## 3 The lessons of forward genetic screens

If we consider GWAS as analogous to genetic screens at a molecular level, the same lessons that apply to model organisms may apply to humans. But what are those lessons, and how can we move forward with them in the age of single-cell biology and artificial intelligence (AI)? We need look no further than the original forward genetic screen in *Drosophila* (Nüsslein-Volhard and Wieschaus 1980). The study found three categories of phenotypes in cuticle pattern, suggesting different organizing processes along the anterior-posterior axis of the embryo. There were “gap mutations,” which resulted in large domains of missing or mispatterned segments, the “pair-rule mutations” which deleted alternating odd or even segments, and the “segment polarity” mutations which disrupted the pattern of denticles (Nüsslein-Volhard and Wieschaus 1980). As paradigm-shifting as this paper was, the authors could not know what lessons awaited. Decades of subsequent experimentation unfolded to reveal how a small number of core molecular pathways can produce infinite biological phenotypes.

### 3.1 Lesson 1: Recovered genes interact with each other

**Figure 1.**
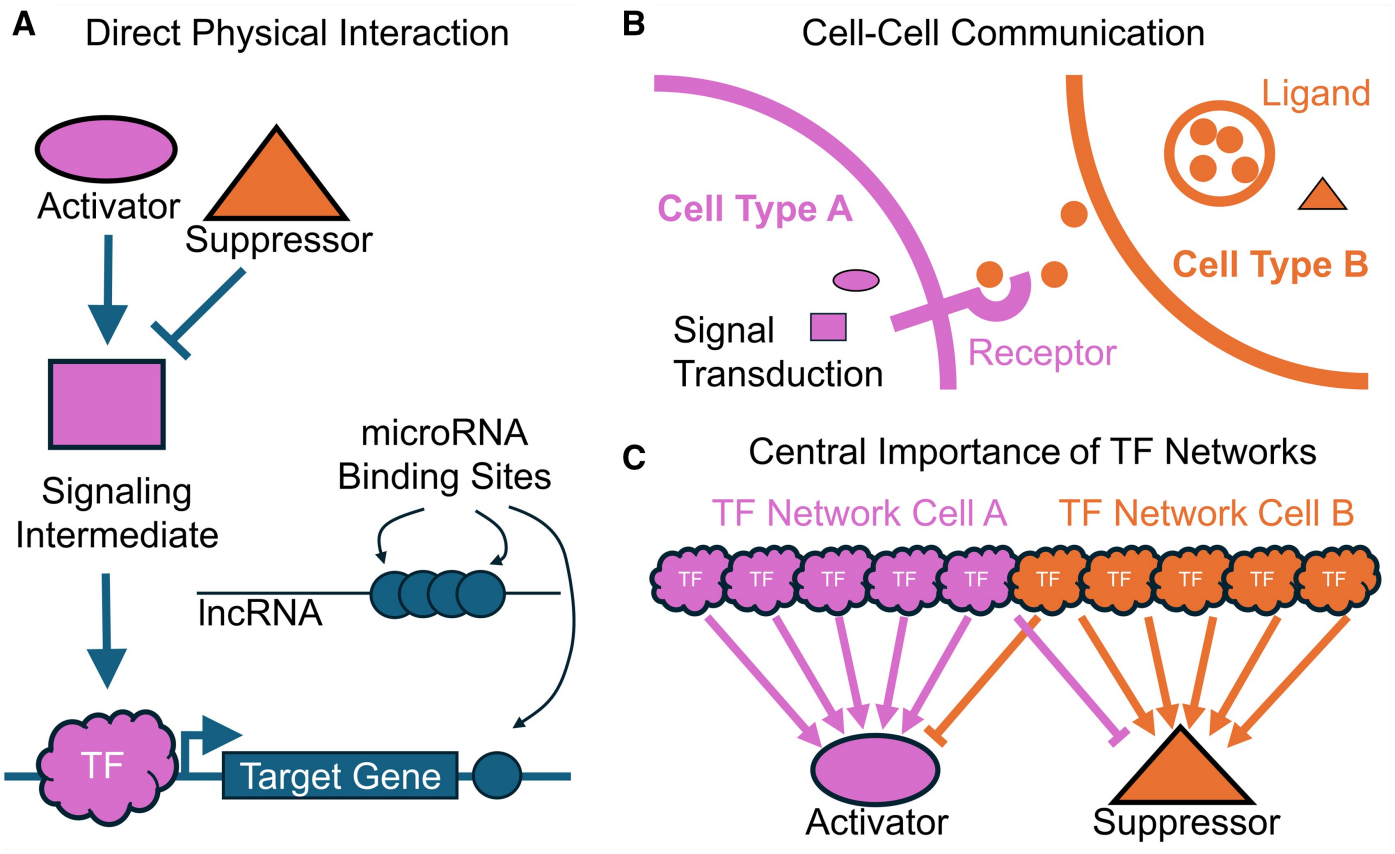
The three lessons of genetic screens in model organisms. (A) Similar cell-specific phenotypes are uncovered from genes that physically interact. These may include members of a signaling pathway, both activators and suppressors, transducers, transcription factors, and their targets. Genes may be regulated indirectly by microRNAs and long non-coding RNAs that act as a sponge for microRNA binding sites. (B) Similar phenotypes may be observed when genes are disrupted from a cell–cell interaction that is required for normal function or to maintain a differentiated state. (C) TF networks make up the bulk of discovery in genetic screens, because many different TFs control the relevant timing and dynamically coded spatial information of a small number of key factors underlying the phenotype.

Genes whose disruption produces similar phenotypes often encode proteins that interact physically to transmit information, or as part of a biochemical pathway. This kind of relationship is shown schematically in Fig. 1A. Strikingly, all 15 genes reported in that original study directly interact with each other to produce normal patterning of the fly embryo. The most famous are *hedgehog* (ortholog of mammalian *Sonic hedgehog Shh*), *patched*, and *cubitus interruptus*, which form, respectively, extracellular ligands, receptors, and signal transducers. These genes were recovered in subsequent screens for other phenotypes [for a modern review of *hh* signaling, see Lee *et al.* (2016)]. *Engrailed*, which is induced by *Hedgehog*, and *wingless*, an ortholog of mammalian *WNT*, each inhibit the other’s expression in their respective cell types, while simultaneously modulating each other’s activities (Basler and Struhl 1994, DiNardo *et al.* 1994). Thus, all five genes operate in a feedback loop to maintain each other’s expression while differentiating along the AP axis to produce a repeated pattern of denticles [for a review of planar polarity, see Lawrence *et al.* (2007)]. The final gene of the segment polarity class, *gooseberry*, is a transcription factor critical for the maintenance of *wingless* expression, thus closing a regulatory loop (Li and Noll 1993). It is astounding that every key player in this pathway was discovered by screening for a particular pattern in the cuticle of larvae, and no less so that the same genes were recovered in subsequent screens for phenotypes that require planar specification of tissues. It is thanks to these genetic screens that we came to understand planar cell polarity as a fundamental axis of multicellular development. Thus, genetic screens efficiently recover genes that act together to produce phenotypes. Some are antagonistic, others activating, but all play a role in ensuring morphogens and intracellular signaling cascades are set in motion at the appointed time and place.

### 3.2 Lesson 2: Heterogeneous cell interactions produce phenotypes

These phenotypes are not self-contained, or in the parlance of developmental biology, not “cell-autonomous.” In fact, there were two morphogens (*wg* and *hh*) and a ligand–receptor pair (*hh/ptc*) in adjacent domains of cells, signaling to each other, limiting the other’s expression and activity. Figure 1B illustrates how adjacent cell types interact to regulate each other’s activity in this manner to produce a phenotype. Where any developmental process occurs—even cancer initiation and progression—patterning, migration, cell death, asymmetric division, and differentiation depend on cell–cell signaling events. It is difficult to imagine how an unbiased screen would fail to produce phenotypes from molecular events occurring in more than one-cell type.

### 3.3 Lesson 3: Transcription factors rule everything

We have yet to discuss the other two categories of phenotypes discovered in the larval cuticle screen, the “pair-rule” and “gap” genes. Every one of these genes turned out to be a transcription factor (TF). The gap genes define broad AP domains, which position the alternating domains of pair-rule TFs. Some TFs repress each other’s expression in adjacent domains. Pair-rule genes provide the context for establishing a repeated pattern of *wingless-Hedgehog* domains. Put another way, the same developmental signaling pathways are controlled by different overlapping sets of transcription factors along the AP axis. The cells in one segment that express *wingless* are under the control of a slightly different regulatory network than in another. Although these cells are very similar at this stage of development, the same genes in different segments respond to differing combinatorial expression of TFs (Schroeder *et al.* 2004). Thus, as illustrated in Fig. 1C, it takes many more TFs than signaling proteins to control every aspect of body pattern and cellular identity. Eleven of the 15 genes recovered in the cuticle screen were TFs, highlighting the importance of this class of genes. By extension, where tissue patterning and differentiation are concerned, we anticipate the central importance of transcription factors.

## 4 Identification of GWAS susceptibility genes poses unique challenges

Most GWAS loci have been interrogated individually. What is the target gene? Considering the extensive parallels to forward genetic screens, is there any good reason that the same principles of interaction, non-cell-autonomy, and transcription factor dominance do not also apply to GWAS? With complex diseases, we should proceed cautiously. For one thing, Alzheimer’s or cancer may represent multiple phenotypes. The celebrated hallmarks of cancer began with 6 in number but have expanded to 14 (Hanahan and Weinberg 2000, Hanahan and Weinberg 2011, Hanahan 2022). Angiogenesis and immune evasion are two distinct phenotypes involving different cell populations, for example. Variants that affect transcriptional enhancers, and even promoters, may impact more than one gene. The *TMPRSS2* gene in prostate cancer, *e.g.* has a risk variant in its promoter associated with oncogenic translocation of the promoter onto *ERG* (Kohaar *et al.* 2020). No direct role for *TMPRSS2* is currently known for prostate cancer susceptibility. Do multigene effects result from genetic associations with enhancers? All six candidate genes at a hypertension locus were shown to affect hypertension in rats (Flister *et al.* 2013). At the *ANKLE1* locus in ovarian cancer moreover, multiple lines of genetic and molecular evidence implicate different genes, creating uncertainty in the “causal” gene (Lawrenson *et al.* 2016, Kohaar *et al.* 2020). It is even possible to have multiple functional variants at a single locus (Corradin *et al.* 2014).

Risk alleles are probably sensitive to certain cell populations and periods of development. One example of this is a susceptibility gene for Parkinson’s disease which is active in neuronal precursors during the growth of the substantia nigra in embryogenesis (Coetzee *et al.* 2016, Prahl *et al.* 2023). Susceptibility genes may also be active in cell types outside the primary affected organ. These perturbations affect normal function, occur early in disease etiology, and are not individually sufficient to cause a phenotype. Rather, they alter probability of developing disease on a small scale relative to and in concert with environmental exposures.

There have been some notable successes for functional interpretation using machine learning approaches and single-cell methods. Integration of single-cell multi-omics data with Alzheimer’s disease (AD) and Parkinson’s disease GWAS implicated the potential importance of oligodendrocytes in AD etiology (Corces *et al.* 2020, Morabito *et al.* 2021). Subsequent studies identified cholesterol metabolism in oligodendrocytes as a key event and led to clinical drug trials (Blanchard *et al.* 2022), thus underscoring the importance of cell-type specific mechanisms.

Due to the great majority of associations being non-coding in nature however, plausible mechanisms are quite varied. Many tools exist for prediction of function, including *HaploReg* (Ward and Kellis 2012, 2016), *funciSNP* (Coetzee *et al.* 2012), *motifbreakR* (Coetzee *et al.* 2015), *VEP* (McLaren *et al.* 2016), and *L2G* (Ghoussaini *et al.* 2021, Mountjoy *et al.* 2021). Most GWAS loci identify more than one likely causal variant due to linkage disequilibrium. Any of these variants might be causal, *i.e.* change the activity of a nearby gene product. Variants occurring within a gene’s coding sequence that result in an amino acid substitution, frameshift, or premature stop codon have a high likelihood of affecting that gene. Variants can occur in the untranslated regions (UTR) of encoded mRNAs, as in the case of *MDM4* in prostate cancer (Stegeman *et al.* 2015) [also reviewed in Steri *et al.* (2018)]. UTRs encode information about mRNA stability and secondary structure, and contain the seed-regions of microRNA binding and regulation (Schuster and Hsieh 2019) (see Fig. 1A). In some neuronal cells, mRNAs are transported to distal regions for local translation (Mofatteh 2020). These RNAs have signal sequences that can be disrupted (Turner-Bridger *et al.* 2020). There is a hierarchy of single nucleotide or small insertion and deletion (indel) variants that can affect gene regulation in myriad ways. Gene expression is activated by the binding of TFs to encoded motifs at promoter sequences. Consequently, variants in promoter sequences have the potential to disrupt these motifs and alter binding and activation of transcription (Lee and Young 2013). The effects of these changes are mediated by activators or repressors of transcription.

We must also consider the possibility that cytosine-guanine dinucleotide motifs (CpGs) are disrupted, resulting in epimutation events which effectively silence one copy of the gene, as has been documented for the *MLH1* promoter for rare private mutations and some common population variants associated with inherited cancers (Hitchins *et al.* 2005). We must consider the effect of CpG disruption on enhancer regions, which bind TFs to regulate initiation of transcription at promoter sequences (Panigrahi and O’Malley 2021). These regions are similarly sensitive to motif disruption and methylation but can be located hundreds of kilobases from the affected promoter, greatly increasing the pool of genes that must be considered potential targets.

Estimates from burden testing of rare variants in GWAS regions suggest a distribution whereby the nearest gene is the likely but not sole best candidate (Weeks *et al.* 2023). Regression analyses of the relationship between methylation of enhancers and nearby gene expression in tumors suggest that enhancers regulate transcription from the nearest gene about 15% of the time (Yao *et al.* 2015, Silva *et al.* 2019). Genes are organized into larger regulatory units called topological association domains, regulated by *CTCF* complexes that separate globular chromatin within which physical interactions most frequently occur [reviewed in Dehingia *et al.* (2022), Del Moral-Morales *et al.* (2023)]. *CTCF* is also a sequence-specific DNA binding protein, whose motif is likewise vulnerable to disruption. *CTCF* motifs at prostate cancer risk variants result in dysregulation or inappropriate expression of a cancer promoting gene (Guo *et al.* 2018b). Therefore any gene in two adjacent topological domains might be a candidate. Similar mutations have been discovered for colorectal cancers (Guo *et al.* 2018a, Liu *et al.* 2018).

Common variants can also be found in non-coding RNAs or their regulatory elements, the ramifications of which are still poorly understood. Non-coding RNAs are known to have important roles in oncogenesis, as they regulate critical pathways like *Hippo/Yap* signaling (Wang *et al.* 2021). There are different types of non-coding RNA with different functions. Some act as microRNA binding sinks, while others interact with proteins as a scaffold or to promote transcription. Current gene prioritization methods and software do not consider these possibilities. Splice site mutations must also be considered (Garrido-Martín *et al.* 2021), and we are only beginning to understand, with the help of machine learning approaches, non-canonical splice sites, some of which are not encompassed by the 5′ and 3′ ends of the gene (Jaganathan *et al.* 2019, Rowlands *et al.* 2019, Rentzsch *et al.* 2021, Zeng and Li 2022). Even synonymous coding mutations have the potential to alter codon usage, which can affect bioavailability of amino acids for tumor growth (Liu *et al.* 2021). To understand how susceptibility genes work together to promote disease, it is necessary to identify as many of these mechanisms as possible.

## 5 On the need for holistic, systems-level interpretation of GWAS

Beyond mechanistic interpretation, we must consider functional links between loci. An early study on the interpretation of prostate cancer GWAS looked at all available genome annotations, untranslated regions, epigenomic data, and TF binding site disruptions. This study identified three pairs of loci whose gene products directly interact, including fibroblast growth factor *FGF10* and its receptor *FGFR2*, Insulin-like growth factor 2 and its receptor (*IGF2* & *IGF2R*), and androgen receptor (*AR*) and its cofactor *NCOA4* (Hazelett *et al.* 2014). These observations echo the three lessons of forward screens: protein–protein interactions, signaling between different cell types, and disruption of transcriptional regulation. But how should one go about creating a model that accounts for and prioritizes such connections?

Because of the importance of heterotypic cell interactions, we cannot simply prioritize genes without also hypothesizing the cell types in which the genes are active. A comprehensive model must account for multicellularity and have internal logic supporting the cell types it proposes. Some methods exist to prioritize genes and cell types. Expression quantitative trait loci (eQTL) correlates expression and genotype in a tissue specific manner (GTEx Consortium 2013, 2015). eQTL and GWAS summary statistics are integrated to prioritize genes by association with complex traits using transcriptome-wide association (TWAS) (Gusev *et al.* 2016, Zhu *et al.* 2016). Multi-marker Analysis of GenoMic Annotation (MAGMA) aggregates gene-level variant association statistics and ontology enrichment for gene sets (de Leeuw *et al.* 2015). scDRS, short for “single-cell disease-relevance score” extends MAGMA by exploring single-cell data sets for enrichment of MAGMA disease signatures in different cell types (Zhang *et al.* 2022). More methods are becoming available to consider diverse evidence sources including multi-omics data (Zhang *et al.* 2022, van Duijvenboden *et al.* 2023) and (importantly) biological interactions between disease loci (Weeks *et al.* 2023). Together these studies provide encouraging signs that the field is grappling with these issues.

### 5.1 The one-gene, one-cell hypothesis

Still, we require a framework to explore discrete mechanistic hypotheses. If we stipulate that there is at least one susceptibility gene at each locus that is no less important than any other, we may consider a streamlined model in which every disease variant mediates its effects via that one gene in one-cell type. Assuming that GWASs, much like forward genetic screens, represent an unbiased survey of critical pathways in disease etiology, then we should consider as part of these models that some loci are functionally related, perhaps even encoding subunits of multi-protein complexes. We should consider models that propose physical or molecular interactions between key cell types. And we might profitably assume that TFs comprise a large fraction if not an outright majority of susceptibility genes. Any model that incorporates these features is consistent with what we have learned from the genetics of model organisms. Therefore, our framework should not consider individual genes as candidates, but rather, *the set of genes and cell types as a whole*, which we may refer to as a “proposal,” *i.e.* the object whose properties must be optimized to meet these various criteria. This one-gene one-cell framework enables a coherent interpretation of the associations as a whole: one that sets forth testable hypotheses at the bench.

## 6 Interpretable AI models of GWAS biology

**Figure 2.**
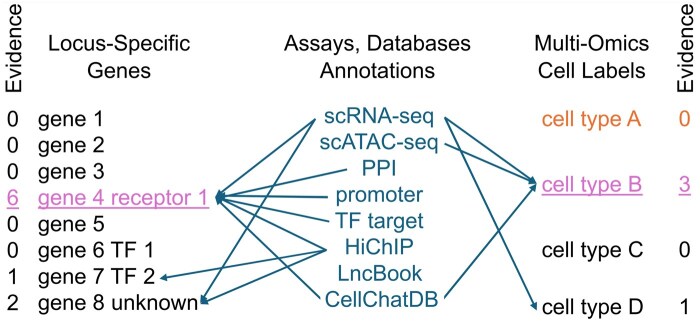
Schematic of how AI might help solve the problem of GWAS functional interpretation. Every genetic association impacts at least one primary target gene (left) in a specific cell type (right). Inference on gene and cell type comes from individual assays, which can affect either gene, cell type, or both simultaneously. The sum of support across all assays and annotations (indicated as “Evidence”) determines the top candidates. This process must be repeated across potentially hundreds of genetic loci, while accounting for and promoting evidence that is consistent with the mechanisms of Fig. 1, with protein–protein interactions being a primary example. Because of the large number of assays, annotations, genes, and cell types involved, the problem is refractory to analysis by relatively simple models.

Ideally we would have a Bayesian model, in which we could ask, “given the observed set of association variants and gene expressions, what are the likeliest susceptibility genes and cell types at each locus that promote disease?” We can treat the entire set of genes and cell types as parameters to be optimized over. It must be appreciated that the proposal space is intractably large. If one assumes a moderate size GWAS with 100 associations, with an average of 10 genes in a 1 megabase region around each locus, we will have to consider 10^100^ proposals. Fortunately, samplers like Stan and modern hardware architectures are fit for this purpose. From there, one requires a likelihood or probability dependent upon the aggregate evidence favoring each gene and cell type of each proposal, as illustrated by Fig. 2. The challenge is writing models that express all evidence sources in a mathematical framework, including hypothetical interactions between loci, which as we have seen, may be a critical feature.

Other algorithms exist to tackle this problem. Large language or foundation models (LLM) might conceivably be used, but a potential limitation is that these models are predictive, based on exposures to prior “ground truth.” The importance of ground truth datasets in establishing the credibility of AI models in other fields is evident. An example of this is protein structure and design, where transformer/diffusion models have achieved high accuracy even without relying on homology, in no small part due to a large fraction (∼17%) of solved protein structures (Tunyasuvunakool *et al.* 2021, Chandra *et al.* 2023). Perhaps such a resource could be created from the large pool of prior genetic screens. This would require overcoming enormous barriers: harmonization of historical screening data (very labor-intensive) across different model systems, organisms, and reagents (*e.g.* mutagenesis, CRISPR, transposons, RNAi). AI and perhaps especially language models could survey the literature to identify gene-phenotype associations to construct gold-standard databases against which to query solution sets. It should be possible to explore functional hypotheses using LLMs, but the challenge remains the lack of synthesis capability. Solutions to these problems are already emerging. Large graph databases such as the recently published AlzKB, known as “knowledge bases,” in which connections between a diverse array of relevant assays, annotations, and drug interactions are mapped, have great potential to speed up discovery (Romano *et al.* 2024). Knowledge Retrieval Augmented Generation ENgine (KRAGEN) builds on this technology, empowering scientists to query and interact with these resources *via* natural language, while monitoring the solutions for consistency (Matsumoto *et al.* 2024).

Genetic algorithms were advanced previously as a solution for integration of complex, overlapping, genetic and experimental evidence in GWAS (Moore *et al.* 2010, Moore *et al.* 2020). Proposals are randomly generated and evaluated along multiple experimental axes or “cases.” A portion of each proposal set with highest “fitness,” defined as a collective score of all cases together, is selected for propagation in subsequent rounds (“generations”). Each case corresponds to a different type of evidence. For example, one case is “missense mutations,” another is “variant in promoter.” Other cases might account for protein–protein interactions (PPIs) between multiple loci, or between a TF at one locus and the promoter of another. Topological associations are measured by Chromatin Conformation Capture (3C) based techniques, which include Hi-throughput 3C (Hi-C) and Hi-throughput Chromatin Immunoprecipitation (HiChIP). Cases can be written to score evidence of enhancer–promoter interaction using these data. They could be combined with single-cell or bulk-seq methods to validate the association. Each of these cases is considered separately, without need to harmonize or represent parameters in the same ordinal space.

These methods have the potential to bear fruit for a couple of reasons. Evaluation of cases in parallel makes it possible to score proposals on disparate evidence such as genome annotations (*e.g.* missense or splicing), cell-type specific expression in single-cell RNA-seq datasets, proximity to epigenomic regions of interest (*e.g.* a cell type specific enhancer) from single-cell assay for transposase-accessible chromatin with sequencing (ATAC-Seq) data (Buenrostro *et al.* 2015), and so on. Each assay provides inference on either the gene target, its likely cell population, or both (see Fig. 2). Ideally, true gene targets and cell types have more than one piece of supporting evidence. Writing cases that weight groups of loci with interacting gene products and logically consistent cell types promotes coherent hypotheses over less viable ones. A recent study of breast cancer GWAS using this approach with normal breast multi-omics data showed promise to integrate diverse data sources into stable solutions (Nguyen *et al.* 2025). Additionally, PPI is one of the most important distinguishing factors between breast cancer GWAS SNPs and matching control variant sets, providing support for the prediction, based on genetic screens, that GWAS loci should involve physically interacting proteins (Nguyen *et al.* 2025).

There are also some significant challenges intrinsic to these methods. It may not be immediately clear what a “successful” solution set is, or whether there are appropriate controls against which to judge it. In the absence of such ground truth solution sets, we may compare predictions against simulated data or random control sets of SNPs, or a gold-standard set as proposed in Zhang *et al.* (2022). As discussed above, AI may be used to expand the catalog of such gold-standard sets (as a proxy for ground truth) at different levels of evidence. Joint modeling of common phenotypes across similar disorders may also increase power, as with both autoimmune diseases and brain-related diseases and traits for example (Zhang *et al.* 2022). Incorporation of similar strategies using more recent attention-based models has the potential to greatly increase the efficiency and accuracy of AI approaches.

## 7 More single-cell experiments from diverse patient populations are needed

At a minimum, a comprehensive atlas of single-cell expression across developmental stages will provide an avenue for unbiased survey of the genome across each GWAS. Disease-specific sets do not capture the majority of loci in our experience, and one hypothesis to explain this disparity is the regulation, growth, and dysfunction of the diseased organ from endocrine events (*e.g.* hormone signaling). It cannot be overemphasized that datasets are required for *normal* tissues to interpret risk biology. Most studies focus on pathologically affected tissue, with normal cells being a byproduct in single-cell datasets. For studying the biology of susceptibility, the focus should be normal cells in normal or control hemispheres. Examples for such studies are preventive surgeries in high-risk patients. Whatever the source, susceptibility genes are likely interacting in multicellular environments long prior to overt manifestation of disease, and it is in this context that we are most interested in their activity. From this perspective, the most high-value datasets are therefore single-cell RNA-seq and -ATAC-seq.

Population variants captured by GWAS frequently disrupt cell type-specific regulatory elements, as evidenced by the tissue-specific functional enrichment of GWAS variants in Roadmap and ENCODE data (Maurano *et al.* 2012, Pickrell 2014, Boix *et al.* 2021). However, bulk sequencing of heterogeneous tissues lacks the specificity to expose the enhancers and very specific cell types they are composed of. In addition, studies are needed on the epigenomics of human populations, as these are known to carry different risks for various diseases and traits. Having a more diverse patient population from which to draw on is critical (Breeze *et al.* 2022). Due to the low sensitivity of current assays, we are more likely to detect disease relevant regulatory elements by including common population variants that differ between and among populations. Study designs that include underrepresented populations should be encouraged, prioritized, and funded for this reason.

## 8 Conclusion: the role of emerging technologies

For functional validation, Clustered Regularly Interspaced Short Palindromic Repeat (CRISPR) technology holds enormous promise. Perturb-seq and related methods use targeted CRISPR deletion of many short sequences in cultured cells in combination with single-cell RNA sequencing (Dixit *et al.* 2016, Datlinger *et al.* 2017). The investigator can rapidly interrogate the effects of regulatory sequences on the transcriptome, potentially capturing up to 90% of GWAS loci.

Finally, spatial transcriptomics data hold promise to test the predictions of our GWAS models *in situ*. We can observe proposal genes and cell types interacting in normal tissue and, importantly, follow their progress as lesions worsen. We can track movement of susceptibility genes and cell types into and out of a lesion as it develops. There are powerful emerging software pipelines to perform these analyses (Martin *et al.* 2022). The other critical technology will be induced pluripotent cell lines (iPSC) established from patients. iPSC enables study of differentiated tissue ecosystems in a dish, where expression and intercellular signaling events can be monitored and validated, their migratory behavior observed, and drug sensitivity assayed. These technologies will be key to discovering and testing new therapies and to interpret GWAS.

Conflict of interest: None declared.
